# Reconciling the contribution of environmental and stochastic structuring of tropical forest diversity through the lens of imaging spectroscopy

**DOI:** 10.1111/ele.13357

**Published:** 2019-07-26

**Authors:** Boris Bongalov, David F. R. P. Burslem, Tommaso Jucker, Samuel E. D. Thompson, James Rosindell, Tom Swinfield, Reuben Nilus, Daniel Clewley, Oliver L. Phillips, David A. Coomes

**Affiliations:** ^1^ Forest Ecology and Conservation Group, Department of Plant Sciences University of Cambridge Cambridge CB2 3EA UK; ^2^ School of Biological Sciences University of Aberdeen Cruickshank Building, St Machar Drive Aberdeen UK; ^3^ School of Biological Sciences University of Bristol 24 Tyndall Avenue Bristol BS8 1TQ UK; ^4^ Imperial College London Silwood Park Campus, Buckhurst Road Ascot Berkshire SL5 7PY UK; ^5^ National University of Singapore 21 Lower Kent Ridge Road 119077 Singapore; ^6^ Centre for Conservation Science, Royal Society for Protection of Birds David Attenborough Building Cambridge CB2 3QZ UK; ^7^ Forest Research Centre, Sabah Forestry Department Sandakan Malaysia; ^8^ Plymouth Marine Laboratory Plymouth PL1 3DH UK; ^9^ School of Geography University of Leeds Leeds LS2 9JT UK

**Keywords:** Beta diversity, dispersal, hyperspectral, LiDAR, neutral theory, niche, tropical forest

## Abstract

Both niche and stochastic dispersal processes structure the extraordinary diversity of tropical plants, but determining their relative contributions has proven challenging. We address this question using airborne imaging spectroscopy to estimate canopy β‐diversity for an extensive region of a Bornean rainforest and challenge these data with models incorporating niches and dispersal. We show that remotely sensed and field‐derived estimates of pairwise dissimilarity in community composition are closely matched, proving the applicability of imaging spectroscopy to provide β‐diversity data for entire landscapes of over 1000 ha containing contrasting forest types. Our model reproduces the empirical data well and shows that the ecological processes maintaining tropical forest diversity are scale dependent. Patterns of β‐diversity are shaped by stochastic dispersal processes acting locally whilst environmental processes act over a wider range of scales.

## Introduction

How so many plant species coexist in tropical forests, despite intense competition for resources, is an unresolved question in community ecology. The answer has far‐reaching implications given the many links between biodiversity and ecosystem functioning (Jucker *et al.,* 2014; Liang *et al.,* 2016). While tropical forests harbour high species richness at small scales (i.e. α‐diversity), they also exhibit high turnover of species over space (i.e. β‐diversity). The notion that environmental complexity underpins β‐diversity by providing alternative niches for species to occupy has long dominated ecological thinking (Grinnell, 1917), but many have questioned whether environmental gradients can supply enough ‘habitat’ niches to explain spatial turnover in tropical biodiversity (Grubb, 1977). More recent theory postulates instead that stochastic dispersal‐recruitment‐death processes within meta‐communities of species with per capita equivalent fitness is sufficient to maintain tropical diversity at local scales (i.e. neutral theory, Hubbell, 2001). The ecological equivalence assumption of neutral theory is often violated in reality, for example by the presence of density dependence facilitating coexistence (Purves & Turnbull, 2010). Nevertheless, near‐equivalence within functional groups (e.g. resulting from life‐history trade‐offs), in combination with weak density dependence, can cause communities to function in apparently neutral ways (Purves & Turnbull, 2010).

Neutral models have successfully matched many biodiversity patterns observed in nature, including species abundance distributions and species‐area relationships (Hubbell, 2001; May *et al.,* 2015), but have been unable to reproduce patterns of β‐diversity over large spatial scales (Condit *et al.,* 2002). Furthermore, when some neutral models are parameterised to reproduce β‐diversity locally, they are unable to reproduce species‐area relationships (May *et al.,* 2015). One explanation for this limitation is that niche and stochastic dispersal processes simultaneously contribute to plant community differences and both must be accounted when modelling β‐diversity (Adler *et al.,* 2007; Chisholm & Pacala, 2010). When tropical communities incorporating niche structure are modelled under entirely neutral assumptions, the model’s estimates of dispersal limitation or speciation parameters may be affected, with the contribution of niches incorrectly interpreted in terms of neutral processes. Indeed, it has previously been noted that niche and neutral processes can be superimposed without strong influence on model predictions (Chisholm & Pacala, 2010), making inference of process from pattern difficult. For instance, species composition exhibits almost universal patterns of spatial autocorrelation that can be caused by the auto‐correlative nature of the underlying environment structuring diffuse niches (Wilson & Keddy, 1986) but equally could be explained by dispersal limitation (Qian, 2009). Disentangling the contribution of niche and stochastic dispersal remains a major challenge, but new opportunities to combine high resolution community structure data with reconstructions of the environment at large scales from aerial surveys might enable us to resolve this problem.

The technology to map biological diversity alongside topographic niche axes across entire regions has only recently become available, enabling ecological theory to be tested on scales unimaginable with traditional field approaches. Airborne imaging spectroscopy, which measures electromagnetic energy reflected off the Earth’s surface in hundreds of narrow wavebands, is a powerful technique for mapping β‐diversity as well as other forest characteristics such as α‐diversity or the functional traits of canopy trees (Asner & Martin, 2009; Draper *et al.,* 2018). Wall‐to‐wall mapping of forests overcomes a major impediment to studying coexistence in the tropics as it enables the drivers of diversity to be analysed at various scales: from the local scales typically sampled by forest inventory plots (0.1–50 hectares), to entire landscapes (i.e. many km^2^). Furthermore, airborne laser scanning can map land topography and 3D forest structure at high resolution, providing further insight.

Working with a hyperspectral image and a 3D laser‐scanning reconstruction of a lowland tropical rainforest in Borneo, we show remote sensing is capable of accurately surveying β‐diversity of canopy trees over a 11.3 km^2^ landscape of hills, valleys and floodplains within which three distinct floristic assemblages of trees have been recognised. By capturing the full extent of spatially autocorrelated β‐diversity, we ask the following questions:

1. To what extent is the spatial structure of the environment (habitat niches) reflected in the spatial structure of β‐diversity?
2. How do stochastic dispersal processes contribute to diversity patterns within landscapes also containing strong niche gradients?

## Materials and Methods

### Site description

The Sepilok Forest Reserve is a *c.* 4000‐ha fragment of lowland dipterocarp‐dominated tropical forest in the state of Sabah, Malaysian Borneo (5°85′N, 117°95′E). The climate is typical of equatorial regions, with a mean annual temperature at the nearby Sandakan airport of 27.6°C and mean annual precipitation of 3481 mm. Three distinct floristic associations are present in our study area (Fig. 2a) growing under differing nutrient limitation: alluvial, sandstone hill and heath. The high‐nutrient alluvial forests, covering the concave relief forms, grow on soils derived from eroding sandstone and mudstone parent material. They form a mosaic with the parallel ridges of the sandstone hill forests formed by the *in situ* weathering of the same bedrock. The nutrient deprived heath forests are located at the eastern part of the reserve and grow on shallow soils derived from a siliceous parent material (Fox, 1973; Nilus, 2004; Jucker *et al.,* 2018). The three communities differ in floristic composition, structure and carbon storage capacity, which indicates strong environmental filtering (Nilus, 2004; Baltzer *et al.,* 2005; Jucker *et al.,* 2018).

### β‐diversity in the field

A network of nine plots, each four hectares in size, was set up in 2000–2001 across the reserve with three replications in each forest type (Fig. 2a). The plots were georeferenced by measuring the position of each corner with a Geneq SXBlue II system, which supports differential correction of GPS signal via the MTSAT Satellite Augmentation System to provide positional error of < 2 m under the forest canopy. The plots were last censused between 2013 and 2015, coinciding with a remote sensing survey of the area. A total of 45 214 stems over 5 cm diameter at breast height were measured and 91% of them identified to species level. Overall, 618 species of 230 genera and 74 families were recorded in the survey. We sub‐divided each plot to four 1 ha plots for our analysis, later removing two of the alluvial sub‐plots outside the remote sensing data extent. β‐diversity of the remaining 34 plots was computed using pairwise Bray‐Curtis dissimilarity (eqn 1) after removing the 9% of stems not identified to species level. While omitting unidentified trees may increase plot similarity, β‐diversity measures are robust to exclusion of such proportion of stems (Pos *et al.,* 2014).

(1) $$d_{\mathrm{jk}}=\frac{\sum \left(|x_{\mathrm{ij}}-x_{\mathrm{ik}}|\right)}{\sum \left(x_{\mathrm{ij}}+x_{\mathrm{ik}}\right)}$$

where *d_jk_* describes the similarity between plot *j* and plot *k* whilst *x_ij_* and *x_ik_* describe the abundance of species *i* in plots *j* and *k* respectively.

### β‐diversity estimated remotely

Imaging spectroscopy is emerging as a powerful technique to map β‐diversity over entire landscapes (Baldeck & Asner, 2013; Féret & Asner, 2014; Draper *et al.,* 2018). We used this spectronomic approach (Rocchini *et al.,* 2004, 2010; Carlson *et al.,* 2007; Laurin *et al.,* 2014) to obtain remote estimates of β‐diversity, independent of field data. A hyperspectral image of the reserve was obtained by a Specim AISA Fenix instrument covering the spectral range between 380–2500 nm in 448 bands with spatial resolution of 1 m^2^. Our approach is detailed in Box 1. Briefly, we start with a raw image of irradiance values, perform quality filtering, dimensionality reduction and β‐diversity estimation, validation against field data and extrapolation over the full extent of the image. We mapped the image onto a high‐resolution digital elevation model obtained by Airborne Laser Scanning (ALS); accounted for effects of scan angles, atmospheric interference and other sources of noise to ensure we only work with high‐quality spectral information that reflects the underlying plant community; we then grouped the image pixels by similarity to reduce the number of non‐independent measurements in our dataset and treated the abundance of each cluster in the plots as ‘species’ for the purposes of computing Bray‐Curtis dissimilarity; we validated the remotely estimated against the field‐estimated β‐diversity and created a dissimilarity matrix covering the whole image extent. A total of 1139 1 ha cells could be placed onto the image, resulting in a matrix with 648 091 pairwise comparisons.

Box 1 **Mapping β‐diversity remotely**

We work with a hyperspectral image of the Sepilok Forest Reserve to derive remote estimates of β‐diversity between 1 ha cells over the landscape. A hyperspectral sensor was mounted on board NERC Airborne Research Facility (NERC‐ARF) operated Dornier 228‐201 aircraft flying at 135 knots in a single survey comprising 13 overlapping flight lines. The survey took place at average altitude of 850 m a.s.l on 5th November 2014 between 10.00 h and 11.00 h local time (solar noon was at 11.51 h). A Leica GPS ground base station was running in the study area concurrently to the flight – information about the plane position was combined with the scan angles of the sensor and the shape of the forest canopy obtained from laser scanning to ensure accurate geolocation of the obtained data with the Airborne Processing Library (Warren *et al.,* 2014). Further details regarding sensor specifications, calibration and georeferencing is available in SI.

First, a series of quality filtering checks were performed to ensure that the hyperspectral data accurately captures the forest signal by identifying and removing potential sources of noise. Data from bad pixels that give wrong or variable readings during calibration (< 1%) was removed. If uncorrected, measurements from those pixels will result in undulating lines following the direction of the aircraft in the mapped image. Artefacts due to atmospheric interference and different scan and sun angles (BRDF effects, Hu *et al.,* 1999) were removed from each flight line using the MODTRAN radiative transfer model (Berk *et al.,* 1999) in ATCOR v. 6.3.2. Pixels illuminated by zenith angles over 50° were removed as the BRDF correction may be unreliable. Pixels with NDVI values < 0.8 were discarded as they indicate low forest cover (Carlson & Ripley, 1997). Each pixel was then brightness normalised following the approach of Feilhauer *et al. *(2010). Bands near the extremes of the sensor spectral range (wavelength below 420 nm and over 2400 nm) are associated with lower signal to noise ratio and were removed prior to further analysis. Similarly, water absorption bands (1350 to 1480 nm and 1780 to 2032 nm) are influenced by atmospheric water vapour and were excluded form our analysis. To improve the signal to noise ratio, the spectral readings in each pixel were re‐sampled by averaging three bands in the visible and the near infrared regions and two bands in the shortwave infrared region. Finally, individual flight lines were combined into a single mosaic by averaging overlapping pixels that passed the quality filtering outlined above, cropping the image to the boundary of the Sepilok forest reserve to exclude urbanised areas and palm oil plantations at its edges.

Second, we employed a spectral clustering approach previously utilised by others (Baldeck & Asner, 2013; Féret & Asner 2014; Draper *et al.,* 2018) to reduce the dimensionality of the data and make it suitable for analysis with intuitive and well established techniques. Image pixels were grouped by the similarity of their spectral reflectance into 250 clusters using the mini‐batch k‐means algorithm (Sculley, 2010). We attempted clustering with values of k ranging between 10 and 800 and noticed that the mean within cluster sum of squares decreases exponentially with increasing values of k up to 250 clusters, after which no further improvement is observed with splitting the clusters (Fig. S2). We could then treat each cluster identity as a ‘species’ for the purposes of computing Bray‐Curtis pairwise dissimilarity between sections of the image. Note that the spectral clusters do not bear a close resemblance to the biological species concept – they are an indirect proxy of the underlying biological diversity that manifests as differential branching patterns, phytochemistry and leaf anatomy that is in turn reflected in the spectral signal.

Third, we used the clusters over the extents of the filed plots to compute a pairwise Bray‐Curtis dissimilarity matrix, independent of the field survey, and correlated this remote prediction to the observed field dissimilarities. The correlation between the two matrices was assessed with a *Mantel’s r* statistic (*Mr*), an extension of the Pearson correlation coefficient (ρ) where significance is assessed with a permutation test to account for the violated independence of observations assumption (Mantel, 1967; Borcard & Legendre, 2012). Finally, after we have validated the ability of imaging spectroscopy to predict β‐diversity over the area of the field plots, we proceeded to compute a dissimilarity matrix over the full extent of the remote survey. We overlaid a 1 ha virtual grid over the hyperspectral image where each pixel is now represented by a cluster identity. We treated each pixel as a ‘species’ for the purposes of computing pairwise Bray‐Curtis dissimilarity for all 1 ha cells with at least 25% spectral coverage. We use this dissimilarity matrix to assess patterns of spatial autocorrelation and partition the diversity into niche and neutral components in this manuscript.

### Landscape environment

#### Habitat types

Previous work in Sepilok has reported that differences in topography and geology across the reserve bias the community composition into three distinct plant associations, that is, create niches (Baltzer *et al.,* 2005; Jucker *et al.,* 2018). We assigned forest types based on a map produced by the Sabah Forestry Department derived from a geological survey of the state. The accuracy of the map was validated by visually inspecting its alignment to topographic features of the landscape measured with ALS and was found to closely match them.

#### Environmental description using ALS

Airborne Laser Scanning (ALS) is an active remote sensing approach that times the returns of light pulses emitted from an airborne sensor and creates an accurate three dimensional reconstruction of the forest floor and canopy. ALS data were obtained during the same flight as the hyperspectral imagery by a Leica ALS50‐II sensor with a field of view of 12°, emitting light pulses with frequency of 83.1 Hz, and a footprint of *c*. 40 cm diameter. The ALS system recorded up to 4 discrete returns per pulse, the data were preprocessed by the NERC‐ARF’s Data Analysis Node and delivered to us as point clouds with an average density of 7.3 points m^−2^. We used the first returns to produce a digital surface model of the forest with 1 m^2^ resolution using LAStools (http://rapidlasso.com/lastools/). Classification of ground points was performed by the *las2dem* module, producing a raster of ground elevation with a resolution of 1 m^2^. The point cloud was standardised by subtracting the ground elevation to produce a canopy height model to 1 m^2^ resolution from the first returns. In summary, we obtained three products from the ALS sensor: (1) an elevational model of the treetops we used for mapping the hyperspectral images; (2) a ground elevation model we used for computing topographic metrics and (3) a canopy height model for computing mean canopy height and tree density maps.

The following five topographic variables were calculated for each one‐hectare cell from the LiDAR‐derived digital terrain model: mean elevation, slope, a topographic position index, a topographic ruggedness index (TRI), and a topographic wetness index. Ruggedness was obtained by calculating the squared elevation difference between each cell and the eight surrounding sells, then taking the square root of the average of these values (Riley, 1999); wetness considers the effect of topography on water flows, and has been shown to correlate with soil depth, organic matter content, pH and available P (Moore *et al.,* 1993). Topographic position corresponds to whether the cell is positioned on a hilltop or a valley (De Reu *et al.,* 2013). In addition, the solar illumination was calculated from LiDAR‐derived digital surface model; solar illumination affects transpiration and photosynthesis rates and was obtained by averaging the direct illumination received by a cell over a year, calculated using the *r.sun* ray‐tracing algorithm in GRASS GIS (Hofierka & Suri 2002). Finally mean top‐of‐the‐canopy height (TCH) was calculated from mean canopy height of each cell and interpreted as a proxy of soil nutrient availability and successional stage.

### Statistical modelling

#### Assessing patterns of spatial autocorrelation

We assessed the extent of spatial autocorrelation of species composition in Sepilok via Mantel correlograms (Mantel, 1967; Oden & Sokal, 1986; Legendre & Legendre, 1998). When constructing a Mantel correlogram, the original diversity distance matrix is paired with a distance matrix based on the Euclidean separation between the plots. The Bray‐Curtis distances are then rearranged following a series of offsets *X* so that the values of the original matrix are shifted to the cells that correspond to position *X* distance apart from their original position. The re‐arranged matrix is correlated back to the original diversity matrix using a Mantel test (Mantel, 1967). For any given offset *X*, a significantly positive value of *Mr* indicates that plots *X* meters apart are more similar than expected by chance and *vice versa*. No spatial autocorrelation is observed when *Mr* values for the distance classes are not significantly different from zero. The number of valid data points to compare reduces as the offset value approaches the distance between the two most distant cells. To avoid this problem, the considered offsets are truncated according to Sturges’ rule (Sturges, 1926).

#### Quantifying niche effects

We used Generalised Dissimilarity Modelling (GDM, Ferrier *et al.,* 2007) to assess the influence of niche structure on β‐diversity, thereby answering our first question: to what extent the spatial structure of β‐diversity is related to environmental niches. GDM is an extension of matrix regression that is able to fit non‐linear relationships between environmental and compositional turnover. GDM partitions variance in β‐diversity into environmental (E), distance (D) and co‐variance (E* × *D, i.e. spatially autocorrelated environment) components. GDM was used to model the influences of (a) forest type and (b) continuous variables derived from ALS on β‐diversity; the forest type factor was included as two orthogonal contrasts. Non‐linear relationships between environment variables, geographic distance and β‐diversity were modelled separately for each forest type. The relative importance of the individual environmental descriptors was assessed by randomising the order of their values in turn and noting the average loss in explanatory power over 50 permutations.

#### Reconstructing landscape β‐diversity with neutral models and generalisations of them

We performed a series of neutral simulations with a colescent model (Rosindell et al., 2008) to uncover the role of stochastic dispersal processes in constructing the diversity of niche‐structured forests. Two sets of simulations were run using pycoalescence (v. 1.2.6 available at https://pypi.org/project/pycoalescence/). In the first set, we treated the whole landscape as a single arena with all interactions between individuals being entirely neutral – we refer to them as ‘forest‐type‐naive’. In the second set of simulations, forest type differences were imposed onto the arena so that interaction within forest types remained neutral, but mixing between them was penalised – we refer to this set as ‘forest‐type‐aware’. While the forest‐type‐aware simulations represent a departure from strict neutral theory to incorporate niche concepts, they remain tractable using spatially explicit simulation methods developed for purely neutral models. We used a combination of 50 different dispersal parameters and seven different speciation parameters in each simulation to obtain a total of 700 ‘virtual’ censuses for comparison against the empirically observed dissimilarity in Sepilok. We detail our modelling approach in SI. Note that finer scale environmental structure such as topography was not included in the forest‐type‐aware simulations. This omission likely leads to inflated values of the speciation parameters in the models that match the empirical observation to account for diversity due to finer environmental structuring.

**Figure 1:**
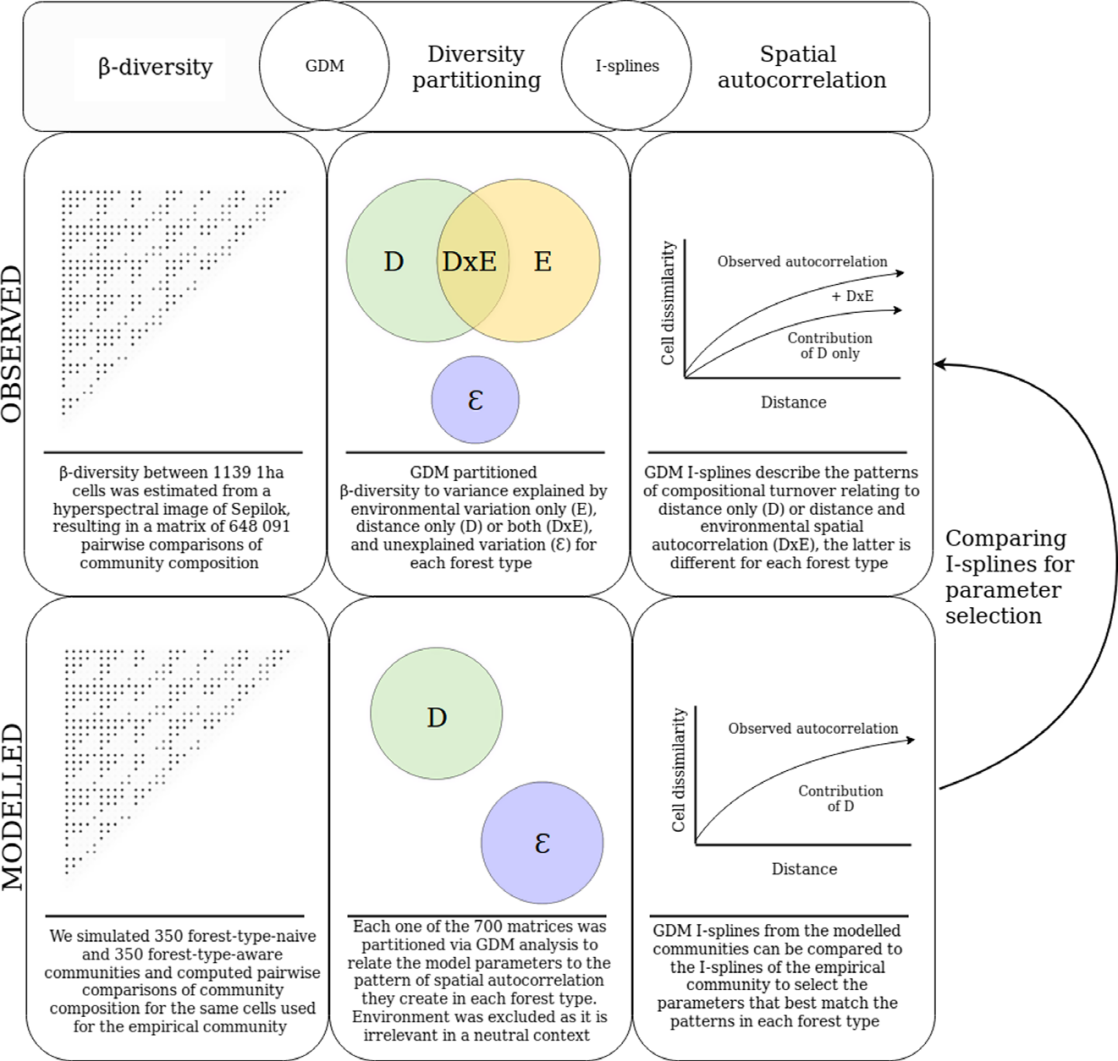
β‐diversity over the Sepilok Forest Reserve in Malaysia, which harbours three forest types growing on soils with varying levels of nutrient availability, was mapped using airborne imaging spectroscopy and a pairwise Bray‐Curtis dissimilarity matrix of 1139 1 ha cells was created. The variation in β‐diversity in each forest type was partitioned as relating to geographic distance (D), ALS‐derived environmental description (E) or both (D×E) alongside unexplained residual variation using Generalised Dissimilarity Modelling ‐ GDM (Ferrier et al., 2007). This analysis relates to our first question: to what extend is the spatial structure of the environment (habitat niches) reflected in the spatial structure of β‐diversity. The GDM analysis outputs a set of I‐splines that describe the relationship between geographic distance (right panel, *x* axis) and cell dissimilarity (*y* axis). The spectral analysis results in two splines of interest, describing the pattern of spatial autocorrelation in composition across each forest type: one of the splines describes the observed pattern (D + D × E) while the other one describes the pattern without the variation that can also be accounted for by the structure of the underlying environment (D only without D* × *E). A series of neutral simulations were performed to test whether the pattern of spatial autocorrelation in composition observed in the spectral survey can be reconstructed under neutral assumptions. A total of 700 communities were modelled and the resulting β‐diversity matrices were analysed in a similar manner to the spectral β‐diversity matrix. As environmental differences are not expected to impact neutral processes, we only used a distance component to partition the diversity of those matrices. Note that variation that lacks spatial structure was not included in our analysis as multiple processes can give rise to it and we cannot partition them with our current analysis, which we elaborate on in the Discussion. However, speciation events in our modelled communities can incorporate species in random positions across the landscape, enhancing between‐plot dissimilarity and accounting for diversity that is otherwise maintained by niche processes. The I‐splines from the modelled communities can then be compared to both splines from the spectral GDM analysis, providing a selection of parameters that best reconstruct the observed patterns in each forest type. One might expect that if a stochastic neutral process maintains patterns of spatial autocorrelation across the reserve, a single parameter set can reconstruct the observed patterns in all three forest types consistently with and without overlaying niche structure as niche processes are not expected to influence neutral interactions. Alternatively, if niche processes influence the pattern of spatial autocorrelation in composition, neutral processes will not be able to reconstruct the patterns driven by the three different environments in Sepilok. This analysis relates to our second question: how do stochastic processes contribute to diversity patterns within landscapes containing strong niche gradients.

Environmental niche filtering and stochastic dispersal act independently of one another and we can therefore expect that the contribution of dispersal in forming patterns of spatial autocorrelation can be reconstructed with a single parameter over the entire landscape. To test if this is the case, we compared the modelled communities, with and without overlaying forest type differences, to the empirical community as mapped from the hyperspectral data. We ran GDM analyses on each modelled community, just as we did for the empirical community, which resulted in a set of I‐splines that describe the pattern of spatial autocorrelation reconstructed by each parameter set. The I‐splines represent a link function between distance and compositional turnover where their slope reflects the rate of change associated with distance and their maximum value relates to the total amount of turnover explained. We compared the I‐splines of the modelled communities from the two sets of simulations to the I‐splines describing the pattern of spatial autocorrelation observed in each of the three forest types, either explained by distance only (D) or by distance and spatially autocorrelated environment (D + D × E). This enabled us to answer the question whether dispersal can account for the patterns of spatial autocorrelation in Sepilok independent of forest type, or whether niche structure is required to explain the observed patterns. Our approach for diversity partitioning, neutral modelling and parameter selection is summarised in Fig. 1.

## Results

The remotely sensed β‐diversity map of Sepilok revealed forest‐type‐specific patterns of spatial autocorrelation in community composition spanning over kilometres – beyond the scales typically considered in field studies. Partitioning β‐diversity to variance explained by distance (D), environment (E) or both (D* × *E), we found that E has variable contribution within the three forest types, but the proportion of β‐diversity explained by D remains constant at ca. 10% of plot dissimilarity in all habitats. The full pattern of spatial autocorrelation in each forest type (D + D* × *E) was successfully reconstructed by a forest‐type‐aware model that utilised a distinct dispersal kernel in each environment. However, when we only required our models to simulate the spatial autocorrelation not explained by the spatial structure of the underlying environment (D only without D* × *E), we found that a single dispersal kernel can reconstruct the pattern of spatial autocorrelation in all three forest types in an entirely neutral forest‐type‐naive model – suggesting that dispersal limitation can act independently of niche structure to create a universal gradient of spatial autocorrelation in community composition, while environmental differences further modify this pattern to enhance β‐diversity.

### Mapping β‐diversity remotely

**Figure 2:**
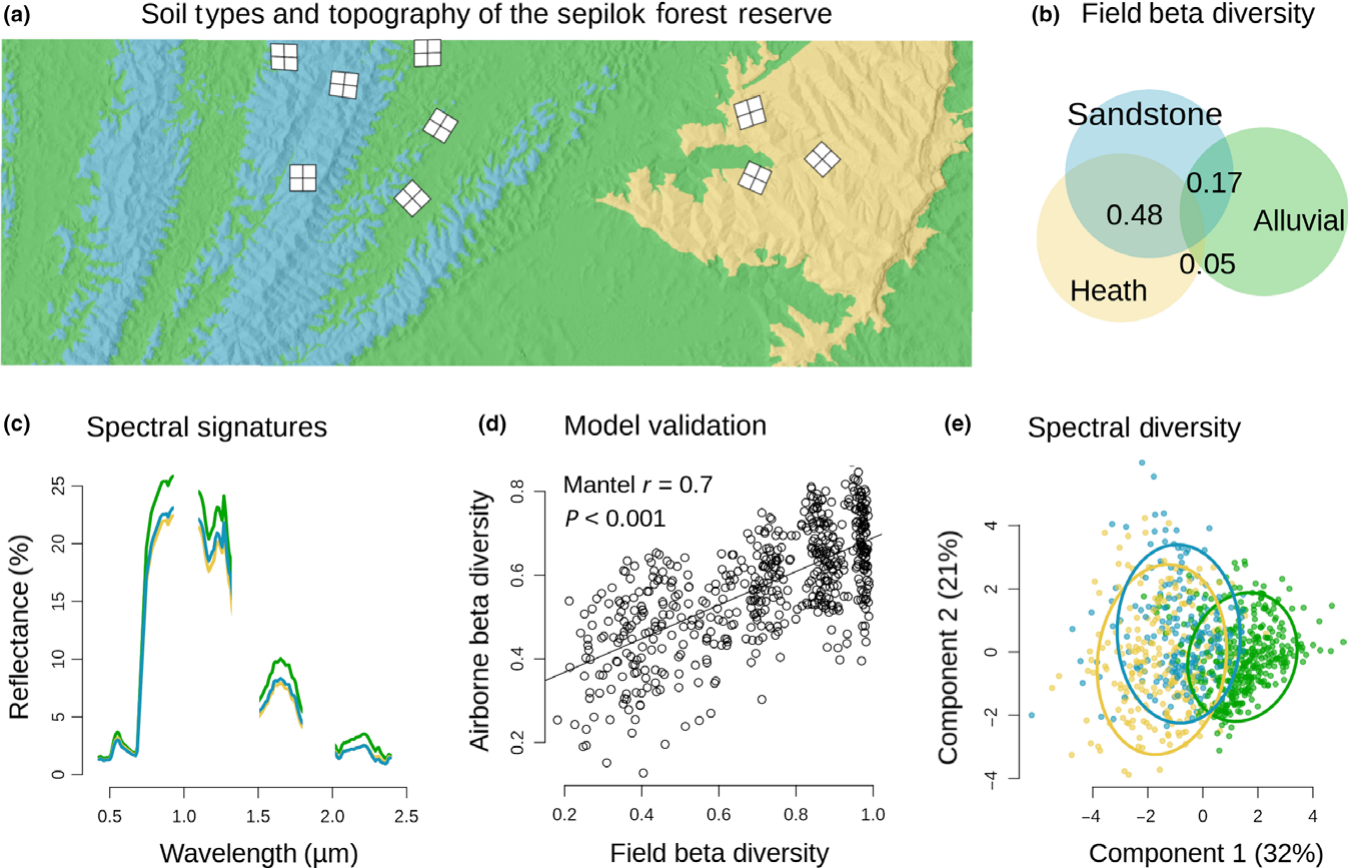
The Sepilok Forest Reserve in Malaysia has three distinct floristic associations (colours) and a hilly topography (shading); nine 4‐ha plots (a) provide field Bray‐Curtis similarity estimates between the three forest types used to create a Venn diagram (b). Airborne imaging spectroscopy measures reflected light in hundreds of narrow wavebands, varying between forest types (c). Non‐supervised clustering was used to map spatial variation (i.e. β‐diversity) of the hyperspectral data; these remotely sensed β‐diversity estimates correlate closely with β‐diversity recorded in field plots (d). Spectral β‐diversity was estimated for the whole landscape by comparing spectra in virtual 1 ha cells laid out in a grid across the reserve. Ordination of spectral clusters among these 1139 virtual plots (e) reveals a similar pattern among forest types to those observed in the field. Ellipses in (e) encompass 80% of observations.

**Figure 3:**
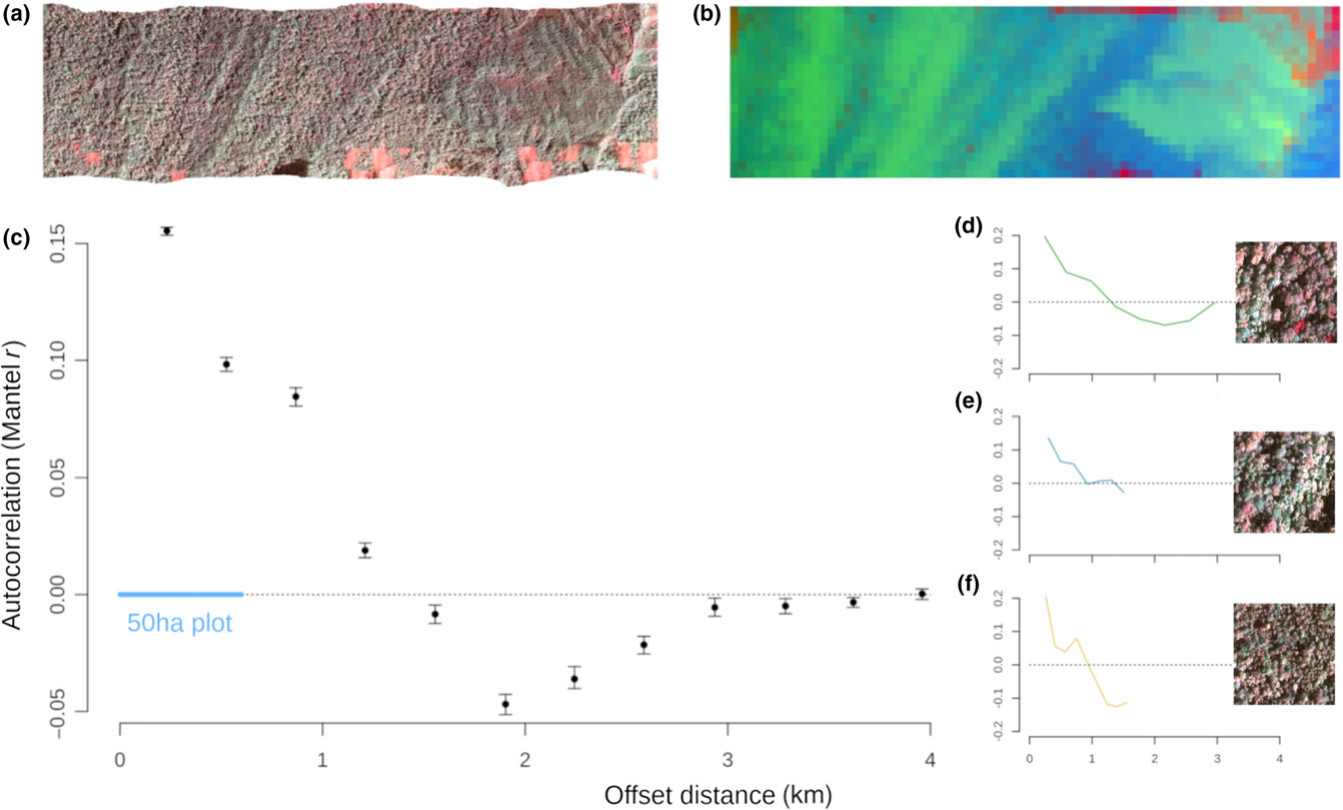
A pseudo‐colour plot of the hyperspectral image of the Sepilok Forest Reserve (a) that has been used to predict β‐diversity between 1 ha cells (b). Pixels in shadow, clouds and other pixels that do not exhibit stereotypical plant spectra were filtered prior to analysis. β‐diversity in Sepilok exhibits patterns of spatial autocorrelation in composition spanning over 3 km (c). Positive values of *Mr* imply communities at any given offset distance apart are more similar than expected by chance and vice versa. Confidence intervals were computed following 500 bootstrap permutations. Distance classes were truncated according to Sturges’ rule (Sturges, 1926). The blue line indicates the extent of the same analysis if plot inventory data from a standard 2 : 1 50‐ha plot were used instead of the landscape survey we conducted and distance classes truncated with the same criteria. Panels on the right present the forest‐type‐specific pattern of spatial autocorrelation for alluvial (d), sandstone (e) and heath (f) forests. Zoomed‐in sections of the hyperspectral image from the three forest types are displayed alongside panels d–f.

Remotely sensed Bray‐Curtis dissimilarity from canopy reflectance signatures correlated with field‐based estimates from 34 one‐hectare inventory plots (Mantel test, *Mr *=* *0.70, *P* *<* 0.001, Fig. 2d), demonstrating the utility of imaging spectroscopy to accurately predict β‐diversity. As expected, forest type was found to have a strong influence on spectral β‐diversity. β‐diversity was found to be lowest between sandstone and heath forests with greater values between each of those forest types and the alluvial communities. This pattern was detected from both the field survey Fig. 2b) and the hyperspectral survey (Fig. 2c,e). The remotely sensed estimates of spectral clusters for the landscape clearly distinguish forest types in ordination space (Fig. 2e) in a manner that is consistent with the field data (Fig. 2b), without relying on the field survey as a training dataset. We detected significant correlation in the community composition of cells as far as 3 km apart (Fig. 3c) where each forest type harbours a distinct pattern of spatial autocorrelation, further emphasising the strong niche structuring of forests in Sepilok (Fig. 3d‐f).

### β‐diversity partitioning

GDM enabled us to partition β‐diversity within forest types to three components: environment only (E), spatial autocorrelation only (D) and spatially autocorrelated environment (D* × *E), where the combination of D and D* × *E relates to the spatially autocorrelated patterns in Fig. 3. Forest types act as broad environmental filters with forest type affiliation explaining 13.9% of β‐diversity. Within forest types, four of the continuous variables had strong influence on β‐diversity: distance between cells and differences in elevation, TRI and mean top‐of‐canopy height. We attempted to account for further variation by considering other environmental variables calculated by high‐resolution laser scanning (i.e. variation in solar illumination, topographic position and wetness index), but these led to little improvement in the models’ explanatory power (*< *1%), so were not considered further.

Exploring the different patterns in the three forest types, we found that environmental variation (E + D* × *E) has variable contribution to β‐diversity (Table 1). Heath forests exhibit the strongest niche partitioning, with E + D* × *E explaining 18.1% of dissimilarity. In particular, taller forest patches have distinct composition compared to shorter patches, complementing strong elevational structuring (Table 1). Alluvial forest β‐diversity appears to be slightly less influenced by niche structuring than heath forests, with E + D* × *E explaining 15.2% of dissimilarity. Mean top‐of‐canopy height ranged from 20 m to over 50 m on these alluvial surfaces and this variation was associated with compositional turnover; elevation and ruggedness were also influential (Table 1). Environmental filtering of sandstone forests is negligible at 1 ha scale, with only 1.3% of β‐diversity explained by environmental variation (Table 1). Universally, the spatial structure of the underlying environment (D × E) was insufficient to account for the spatial auto‐correlation of the composition in Sepilok (D + D* × *E) in any of the forest types.

**Table 1:** Output of GDM partitioning of β‐diversity in the three forest types in Sepilok. β‐diversity is partitioned into variance explained by distance between plots (D), environmental variation (E) or both (D × E). The environmental description (E) in each forest type consists of mean top‐of‐canopy‐height (TCH), terrain ruggedness (TRI) and mean plot elevation. Some of the variance explained by E could not be allocated to a particular component due to co‐variance

	Partition	Alluvial	Sandstone	Heath
Diversity partitioning between forest types	13.9%	–	–	–
Diversity partitioning within forest types	D	11.2%	10.4%	11.6%
	D×E	8.2%	0%	10.4%
	E	7%	1.3%	7.7 %
	**Total explained**	**26.4%**	**11.7%**	**29.7%**
Constituents of E	TCH	4.5%	*< *1%	9.4%
	TRI	2.2%	*< *1%	*< *1%
	Elevation	2.7%	*< *1%	7.4%
	Co‐variance	5.8%	0%	1.3%

## Contribution of stochastic dispersal

**Figure 4:**
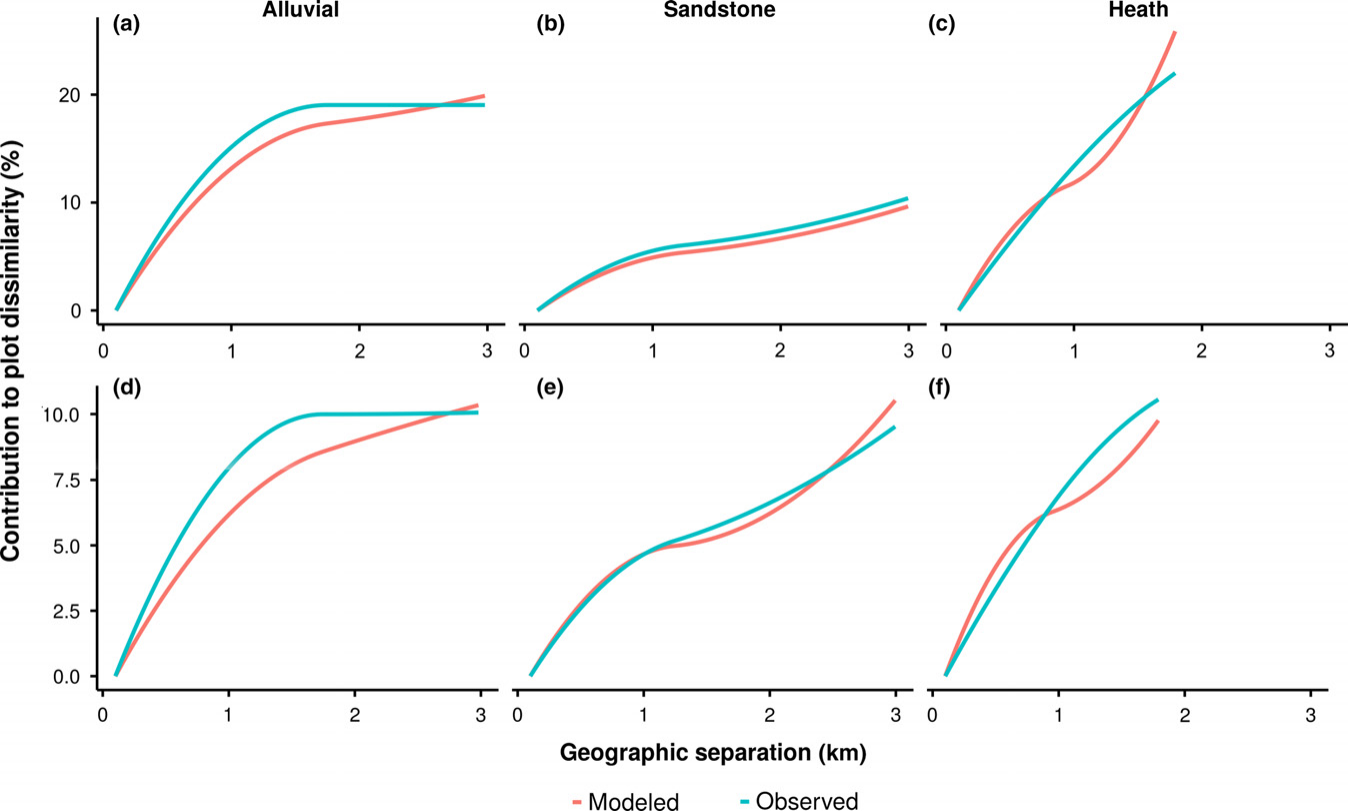
GDM I‐splines representing the compositional spatial structure in Sepilok as observed by the hyperspectral sensor (blue) and modelled by the closest matching neutral simulation (red). Panels a‐c represent the full pattern of spatial autocorrelation (D + D × E) matched by forest‐type‐aware models, each requiring individual parameter set of speciation (ν) and dispersal kernel (σ, conceptually analogous to the mean dispersal distance and τ, conceptually similar to the fatness of the tail) (alluvial: ν = 1^−5^, σ = 10*.*339735, τ = 11*.*650412; sandstone: ν = 1^−8^, σ = 15*.*472447, τ = 1*.*674365; heath: ν = 1^−4^, σ = 9*.*548889, τ = 12*.*820312). Forest‐type‐naive simulations were able to match the spatial pattern that does not covary with environmental structure (D only without D* × *E) using a single parameter set (ν = 5^−5^, σ = 5*.*925375, τ = 8*.*884978) for the whole landscape (d–f).

Stochastic dispersal, especially if regarded as a neutral process, can be expected to act independently of niches in creating patterns of spatial autocorrelation in community composition. Reconstructing those patterns, however, required us to create forest‐type‐aware simulations that departed from strict neutral theory using three forest‐type ‘niches’ with restricted mixing and the ability to maintain differing dispersal kernels. Only then could we match the observed patterns using distinct parameter sets for each forest type (Fig. 4a–c), which is surprising given that if the patterns were genuinely the result of a neutral process, they would not be differentiated along niche boundaries. Repeating the same parameter selection against the D only component of the spatially autocorrelated pattern from the forest‐type‐naive simulations, which do not take into account the presence of forest‐type differences, revealed that the same model parameters can reconstruct the pattern without the need of restricting mixing between the environments (Fig. 4d–f). This is consistent with a process that operates independently of niche structuring.

## Discussion

### Reconciling niche and stochastic processes

Disentangling the influences of environmental filtering and neutral processes on tropical diversity is an enduring challenge (Adler *et al.,* 2007; Chisholm & Pacala, 2010; Purves & Turnbull, 2010; Chisholm *et al.,* 2014). Imaging spectroscopy is emerging as a powerful tool to map tropical forest diversity by detecting differences in reflectance patterns between canopy species with different foliar chemistry and branching architecture (Baldeck & Asner, 2013; Fe´ret & Asner, 2014), but few studies have used this technique to test ecological theory (e.g. Draper *et al.,* 2018). This paper capitalises on advances in remote sensing technology and powerful spatial‐analysis tools to map β‐diversity and test ecological theories of coexistence in diverse tropical forests. We show that multiple environmental gradients act as niche axes along which biodiversity is structured and that, once these environmental contributions were accounted for, residual spatial variation was reconstructed with a neutral model using a single dispersal parameter for contrasting forest types. This is indicative of niche structuring that gives rise to landscape β‐diversity patterns while local dispersal limitation enhances diversity independent of niches.

Much emphasis has been placed on using purely niche or neutral frameworks to explain diversity patterns of tropical landscapes with more recent efforts to reconcile the two theories (Wennekes *et al.,* 2012). It has long been evident that environmental differences are insufficient to explain the high species richness of tropical forests (Grubb, 1977) even though niche theory has been enhanced with over 100 theorised processes that limit or delay competitive exclusion, enabling species to share similar niche space (Wright, 2002). An alternative approach envisioned biodiversity as arising from stochastic events such as colonisation‐extinction equilibrium (MacArthur & Wilson, 2001) or speciation‐extinction equilibrium (Hubbell, 2001), successfully reconstructing complex biodiversity patterns in a simple framework. While some have suggested that neutral models provide a null hypothesis for detecting niche presence, others have questioned the usefulness of such approach (Gotelli & McGill, 2006) – niche processes can superimpose with neutral models (Chisholm & Pacala, 2010), making inference from pattern difficult. Furthermore, neutral simulations suffer from incompatibility between regional and local scale fits: they considerably under‐represent the number of rare species (O’Dwyer & Cornell, 2018) while also not accounting for over‐dominance, which was shown to be reliant on habitat restrictions (Pos *et al.,* 2019). An emergent way forward is to view tropical biodiversity as a product of the interaction between deterministic niche processes and stochastic neutral processes. We follow this idea in our analysis by addressing the questions of how stochastic and niche processes interact to maintain tropical diversity over a continuous landscape and successfully partition the contribution of limited dispersal and spatially autocorrelated environment.

### Environmental influence on β‐diversity

Topographic and soil differences have long been recognised as key drivers of species distributions and biological diversity within tropical landscapes at a range of scales (Ashton, 1964; Baillie *et al.,* 1987; Potts *et al.,* 2002; Russo *et al.,* 2005). Working in northern Borneo, Russo *et al. *(2008) showed that soil‐related habitat variation affects the growth rate of regenerating trees, but also the competitive interactions between them and the forest canopy: it is the combination of these processes that filter species. Our hyperspectral analyses confirm the importance of forest type and topography as environmental filters that generate β‐diversity. Because of the large extent of our analyses (1139 ha), and the high resolution of our topographic maps, we were able to disentangle the contribution of several processes.

Heath forests exhibit the greatest niche structure at 1 ha scale ‐ mean elevation and TCH are indicative of catena effects in soil nutrient availability across the sloped bedrock, which is reflected in the species composition: previous studies distinguished large‐crowned forests at lower elevation, dominated by *Shorea multiflora*, from small‐crowned forests in ridges, dominated by *Tristaniopsis merguensis* (Sabah Forestry Department, 2002). Alluvial forests’ β‐diversity is related to terrain ruggedness, elevation and TCH. The lowlands have alluvial areas within which lie scattered hillocks (rising < 30 m from the plain) which differ markedly in flooding regime (Born *et al.,* 2014). In the temperate rainforests of southern New Zealand, the alluvial soils contain considerably more phosphorus than the older surfaces through which they cut, even though the height difference is only a few metres, and support different tree species (Coomes *et al.,* 2005). Sentinel trees in the alluvial habitat, some 80 m tall, tower over a dense subcanopy (Coomes *et al.,* 2017), under which regeneration is presumably very slow (see Coomes *et al. *(2005) for an analogous example). Patches that have been disturbed, followed by closing of the canopy gap and delayed establishment of tall emergent trees, are likely picked up in our analysis of canopy height differences.

Somewhat surprisingly, we observed little niche differentiation in the sandstone forests in our survey. These forests are found on steep hillsides, often with highly variable topography at a sub‐hectare scale (all environmental variables are more variable within the 1 ha plots than between them, see Fig. S4), so niche differentiation may occur at scales too small to detect with the airborne spectrometer. In fact, such finer scale niche differentiation is likely happening in the other two environments, which we discuss later. We found that β‐diversity persists even in absence of environmental differences: the proportion of spatially autocorrelated composition that does not relate to environmental differences (D) is surprisingly consistent between forest types, causing us to conclude that habitat niche differentiation is insufficient to account for tropical biodiversity patterns (Grubb, 1977; Wright, 2002).

### Stochastic processes enhance tropical biodiversity independent of niches

The Sepilok Forest Reserve harbours distinct niche structure where forests as tall as 80 m grow on nutrient rich periodically flooded alluvial soils and transition to short densely packed unstratified forests on a nutrient poor well drained soils (Jucker *et al.,* 2018). We show that each forest type is characterised by a distinct pattern of spatial autocorrelation (D + D* × *E). Traditionally, ecologists have been cautious when interpreting the covariance component (D* × *E). While it is notable that the variation assigned to D* × *E could arise from either niche (Wilson & Keddy, 1986) or stochastic (Qian, 2009) processes, neutral processes are expected to remain constant irrespective of the environment. When we attempted to reconstruct the full pattern of spatial autocorrelation (D + D* × *E), we were only successful when we made our models aware of forest differences and parameterised them differently for each environment – a clear niche consideration. On the other hand, the distance only component (D) of our GDM models is remarkably consistent between environments and was reconstructed independently of niche structure in Sepilok (i.e. with single parameter set across forest types).

While species are often not functionally equivalent (Dalling *et al.,* 1998; Slot & Poorter, 2007; Kunstler *et al.,* 2016) and local environments filter plant community composition by favouring certain functional groups over others (Grinnell, 1917; Kraft *et al.,* 2008; Paine *et al.,* 2011), as environments become increasingly similar, species can interact in an apparently neutral fashion, enabling coexistence and enhancing diversity. We showed that neutral models within forest types can be individually parameterised to match the observed patterns within each forest type but not over the entire landscape. This is consistent with previous attempts to reconstruct tropical diversity under neutral assumptions that were successful at small scales (May *et al.,* 2015) but not on large scales (Condit *et al.,* 2002). We argue that those disparities are caused by superimposed niche and neutral processes that create biodiversity patterns (Condit *et al.,* 2002; Chisholm & Pacala, 2010) and we successfully reconstructed patterns of spatial autocorrelation in neutral context once niche effects were excluded. To our knowledge, ours is the first study to partition the interaction between limited dispersal and niche gradients over a continuous landscape.

### Residual variation: causes and consequences

Four main processes can give rise to the non‐spatially structured residual variation in our GDMs: unmeasured environmental variability, density‐dependent processes, stochastic demographic processes and errors associated with hyperspectral sensing. Within‐plot environmental filtering is likely to be important – discrete environments within the 1 ha plots we consider are likely to filter the community, enhancing local diversity. Environmental heterogeneity is lowest in heath forests and highest in sandstone hills, which is reflected proportionally in the amount of residual variation in our GDM models (ϵ). Furthermore, niche axes undetectable by ALS may operate at larger scales. The neutral models reconstruct 1 ha plots as single homogeneous environments and any environmental variation that enhances diversity below that scale is falsely incorporated in the biologically unrealistic speciation parameters in the models to match the diversity otherwise maintained by niches or long‐distance dispersal. There is some empirical support that negative density dependence enables coexistence both at local (i.e. Janzen‐Connell interactions, Hammond & Brown 1998) and larger scales to reduce dominance of abundant species (Hubbell, 1986). Those processes would superimpose over the finer scale niche structuring but are currently impossible to partition with our approach. Stochastic processes may also play a role in accounting for the unexplained residual – it has been noted previously that under‐story plants rarely compete directly with one another due to the manifestly asymmetric competition with canopy trees (Wright, 2002). Therefore their interactions with each other can be effectively ignored. Furthermore, when environments become sufficiently similar, species with similar competitive strategies can interact in nearly neutral manner, enabling stochastic processes to structure plant diversity. Finally, sampling artefacts due to sensor error, visible wood and leaf orientation may remain in the data and manifest as random differences between plots despite our best efforts to account for them; the canopy spectral signatures can also be impacted by lianas and epiphytes, which are not recorded in the ground survey.

We could have used smaller than 1 ha cells for our analysis, which would have enabled us to incorporate more of the finer environmental variation. However, this would lead to undesirable edge effects with tree crowns rooted in one cell influencing reflectance in adjacent cells. Therefore, disentangling the contribution of sub‐hectare processes to species distributions over entire landscapes is not yet possible, but technological and methodological advances will soon enable such analysis. Hyperspectral and laser scanning sensors are now sufficiently compact to mount on a UAV. Three dimensional hyperspectral images are already being used to classify individual tree species (Nevalainen *et al.,* 2017) and the high point density and low footprint of UAV‐mounted laser scanners can detect individual stems, providing an intimate link between a tree crown and the environment in its rooting position (Wallace *et al.,* 2012; Chisholm *et al.,* 2013; Wallace *et al.,* 2014). Such maps have the potential to provide individual tree resolution nearly comparable with field inventory plot data without sacrificing spatial extent, underpinning analysis capable of fully accounting for niche separation axes, both spatially and non‐spatially autocorrelated. Future studies can build on the concept we introduced here and use more extensive environmental maps to constrain niche‐neutral models to further understanding of the drivers of tropical diversity.

## Conclusions

Our work shows that tropical forests exhibit patterns of spatial autocorrelation spanning much farther than what can be detected using even the largest of field inventory plots (*c. *50 ha in size) with distinct forest types characterised by distinct β‐diversity patterns. Our analysis confirms that β‐diversity is structured by environmental factors, and that spatial autocorrelation in composition arises, in part, from the spatial organisation of the environment itself. Only after accounting for the effects of environmental filtering could we quantify the contribution of limited dispersal and use entirely neutral models to reconstruct it.

## Authorship

BB, DAC, TJ and DFRPB came up with the idea for this paper; DAC co‐wrote the grant and organised the remote sensing survey; BB, DC and TS processed the hyperspectral and LiDAR datasets. DFRPB, RN and OP contributed field data. SEDT and JR performed the neutral simulations. BB conducted the data analyses and wrote drafts of the manuscript under DAC’s supervision. All authors contributed to the development of the final version of this manuscript.

## Data Availability

Airborne data are available via the CEDA archive (project code MA14/21); plot data is archived on forestplots (Lopez‐Gonzalez *et al.,* 2011) (codes SEP‐03, 04, 05, 07, 08, 09, 10, 11, 12) and from the Figshare Repository: https://doi.org/10.6084/m9.figshare.8427998.v1.
