# Supplementary material for: Reconciling the contribution of environmental and stochastic structuring of tropical forest diversity through the lens of imaging spectroscopy
Source: Ecol Lett. 2019 Jul 26;22(10):1608–19. doi: 10.1111/ele.13357 (PMC6852337; doi:10.1111/ele.13357)
Supplement: Supplementary file 1 [file ELE-22-1608-s001.pdf]

Supplementary information
Reconciling the contribution of environmental and stochastic structuring of tropical forest diversity through the lens of imaging spectroscopy

Boris Bongalov, David F.R.P. Burslem, Tommaso Jucker,
Samuel E.D. Thompson, James Rosindell, Tom Swinfield,
Reuben Nilus, Daniel Clewley, Oliver L. Phillips, and David A. Coomes

β-diversity of canopy trees

Airborne imaging spectroscopy only captures signal from the forest canopy. This is why we interpret our results as relating to β-diversity of canopy trees in this manuscript. However, when we validate the ability of imaging spectroscopy to estimate β-diversity, we use a field inventory dataset that covers all stems with DBH over 5 cm. We choose to do so as it enables the Reader to compare the remote estimates of β-diversity that we report with what is usually calculated from a full inventory plot survey. As our field dataset is characterised by a strong relationship between canopy trees and the full inventory plot survey, the fit we present in Fig. 2 is not influenced considerably by inclusion of under-story vegetation.

To illustrate this relationship between canopy diversity and full plot diversity, we performed two types of analysis. First, we applied an allometric relationship between tree DBH and crown area, which enabled us to estimate which trees form the canopy. The approach is detailed in Jucker *et al.* (2018). Briefly, for each 10x10m cell of our field plots, we kept the largest trees in the cell until the crown area reaches the cell area, discarding trees with lower DBH as under-story vegetation. We estimated Bray-Curtis dissimilarity with this reduced survey of canopy-only trees and related it to Bray-Curtis dissimilarity from the full survey using a Mantel test (Mantel, 1967). Second, we weighted the trees in the full survey by DBH so that larger stems would influence β-diversity measurements proportionally more than smaller stems. Again relating this modified β-diversity measurement to Bray-Curtis distance from the full field

survey.

We find that all three ways to calculate β-diversity from the field survey converge on similar estimates. Filtering under-story vegetation leads to estimates of β-diversity that are highly correlated to the estimates from the full survey (Mantel test, *Mr* = 0.99, p=0.001). The same is true for weighing trees' contribution to β-diversity by DBH (Mantel test, *Mr* = 0.99, p=0.001).

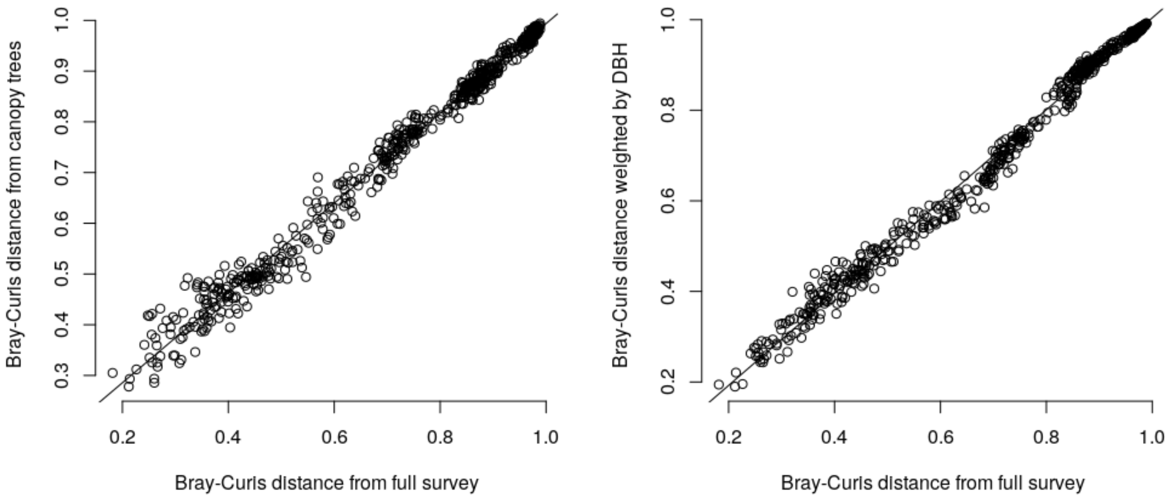

Supplemental Material, Figure S1: β-diversity calculated from the full field inventory survey (x axis) relates well to both β-diversity of canopy trees (left panel) and DBH-adjusted β-diversity estimates (right panel).

Hyperspectral sensor specification, calibration and data filtering

We used an airborne hyperspectral image of the Sepilok Forest Reserve obtained by a Specim AISA Fenix sensor. The sensor contains two photosensitive cells: one covering the visible and near infrared region and the other the shortwave infrared region to provide spectral range between 380 nm - 2 500 nm. All wavelengths are captured by shared optics configuration and produce a single continuous image. VIS-NIR light is captured with spectral resolution of 3.5 nm and SWIR light with resolution of 12 nm. Further specification is available on the manufacturer website (https://www.specim.fi/products/aisafenix-hyperspectral-sensor/). The sensor was mounted onto a Dornier 228-201 aircraft operated by the NERC Airborne Research and Survey Facility (NERC-ARF).

NERC-ARF performed a wavelength calibration with known wavelengths emitted from Hg-

Ar, He, H, Kr, O, Ne and CO_2 lamps. A radiometric calibration was also performed by viewing an integrating sphere that meets the National Physical Laboratory standards. The integrating sphere provides known radiance at each wavelength that was used to produce a calibration curve applied to data collected with the sensor. Radiometric calibrations performed two months apart (December 2013 and February 2014) demonstrated that the sensor is stable (average 0.15% change) with the exception of SWIR water absorption bands (2% change), a part of the spectrum not used in this study. The raw irradiance data (amount of energy reaching the sensor) was delivered to us by NERC-ARF alongside a data quality mask flagging bad pixels in the sensor ($<1\%$) and synced but not applied flight information (i.e. data processing level 1). We applied the data quality mask and then georeferenced the image using the flight positional information, sensor field of view vectors and a high resolution digital elevation model of the tree canopies obtained by a ALS sensor during the same flight. As the field of view (FOV) of the hyperspectral sensor (18°) is wider than the FOV of the ALS sensor (12°), some of the image falls outside of the elevation model extent. We used the satellite-derived ASTER elevation model to patch the high resolution ALS model and provide a surface for the hyperspectral image to map onto. Pixels mapped onto coarse resolution satellite-derived surface models were later removed from our analysis as BRDF correction without good representation of the imaged surface is unreliable.

The mapped image contains artefacts due to scan and sun angles (BRDF effects) and atmospheric interference (aerosols, water vapour, etc.). We corrected for those artefacts using the MODTRAN radiative transfer model (Berk *et al.*, 1999) as implemented in ATCOR v. 6.3.2. The atmospheric correction standardised the raw irradiance values to reflectance values, making values from different flight lines comparable. We removed pixels illuminated over zenith angles over 50° as BRDF correction might be unreliable. We also applied an NDVI filter (NDVI < 0.8) to remove pixels that do not exhibit a stereotypically plant signature, which might indicate they are coming from a non-forested patch or there has been a problem with obtaining or correcting the signal. Pixels with similar spectral signatures but different brightness might appear distinct on the image, hence we applied a brightness normalising function as described by Feilhauer *et al.* (2010). We also filtered spectral bands from each pixel that passed the quality filtering criteria above: the bands near the extremes of the sensor ranges are characterised by lower sig-

nal to noise ratio (wavelengths below 420nm and over 2400nm) and water absorption bands (1350 to 1480 nm and 1780 to 2032nm) are influenced by atmospheric water vapour. Cells centred on a particular wavelength are influenced by adjacent wavebands; we took advantage of this autocorrelation between adjacent bands to spectrally resample the pixels to preserve the signal while reducing the random fluctuations in the signal. We averaged three bands in the visible and the near infrared regions and two bands in the shortwave infrared region. Finally, individual flight lines were combined into a single mosaic by averaging overlapping pixels that passed the quality filtering outlined above, cropping the image to the boundary of the Sepilok forest reserve to exclude urbanised areas and palm oil plantations at its edges

Selection of number of clusters for dimensionality reduction

We used a modified version of the k-means clustering algorithm (Sculley, 2010) to group pixels by similarity. The k-means algorithm divides the observations into k number of groups, such that within-cluster sum of squares is minimised and between-cluster sum of squares is maximised. We tested a range of values for k and noticed that within cluster sum of squares decreases as the value of k increases, up to 250 clusters, when virtually all the variation in the data is captured by the clusters. The value of 250 for k was chosen for our analysis (Fig. S2).

Environmental maps of Sepilok

We used airborne laser scanning to map environmental variation over the Sepilok landscape. The laser scanning sensor sends pulses of light down the forest canopy and times their return. The first returns come from the forest canopy and enable us to compute a digital elevation model; some pulses reach all the way down to the forest floor and enables us to map the topography of the forest floor. Subtracting the elevation model from the terrain model provides us with a model of tree heights. We used this data to compute a number of metrics as described in the main text and visualised below (Fig. S3).

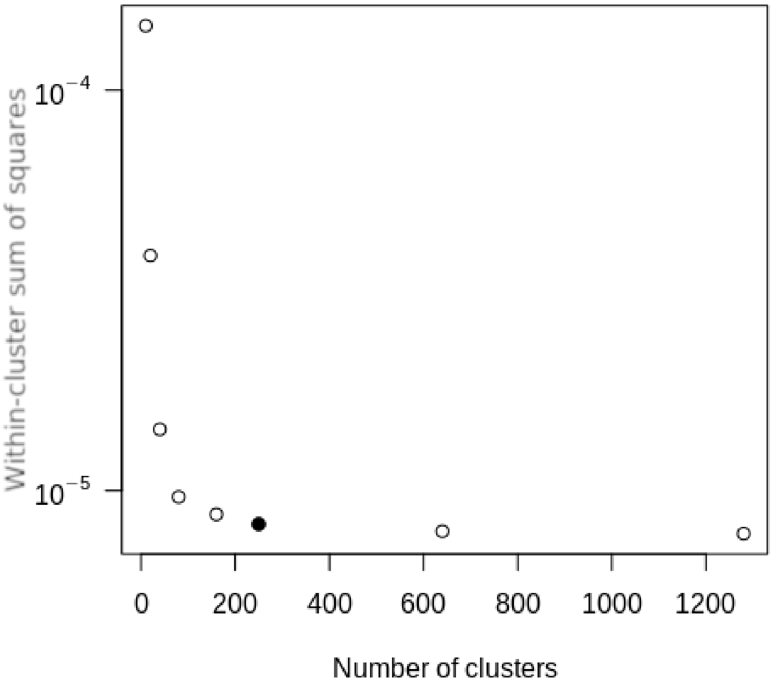

Supplemental Material, Figure S2: Mean within cluster sum of squares decreases with increase of the number of clusters up to 250 clusters (black circle), after which little reduction is observed with splitting the clusters further.

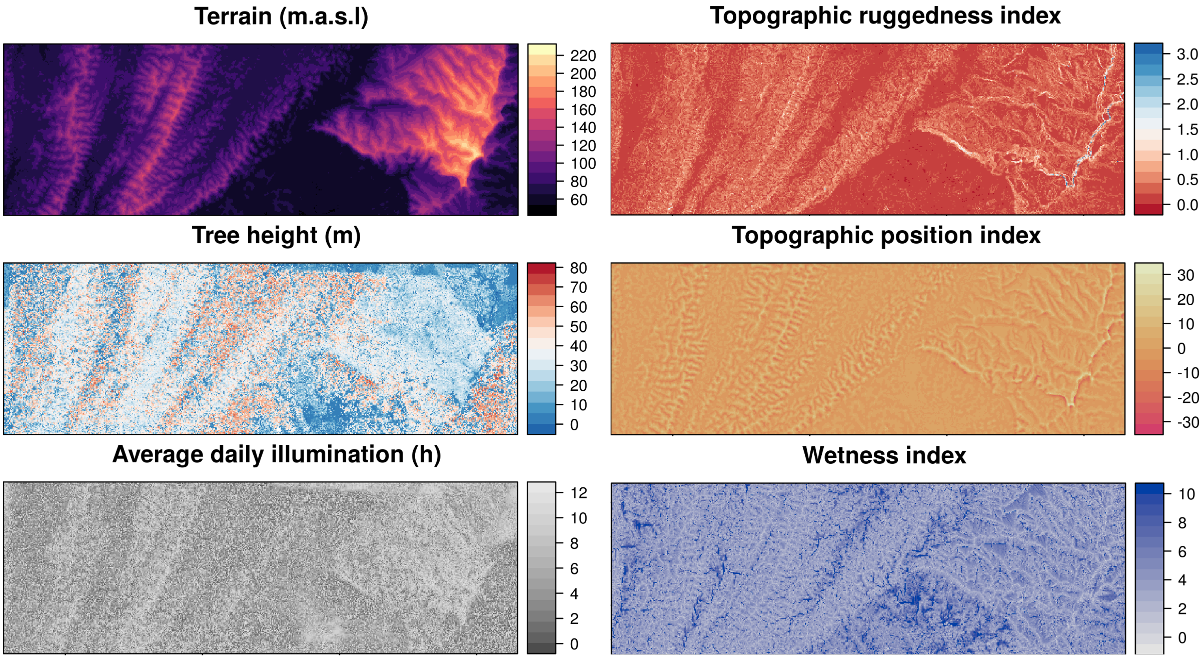

Supplemental Material, Figure S3: Maps of ALS-derived environmental descriptors

Environmental variability between and within cells

We used generalised dissimilarity modelling to relate environmental to compositional turnover. We found that the environmental variables that had the greatest influence on compositional turnover were the ones that vary more between cells than within cells. The figure below (Fig. S4) illustrates within and between cell environmental variation. The cumulative distribution of mean plot environment is represented by a black line - steep slopes relate to little difference between cells and shallow slopes to more variation between plots. Within plot variation is represented by horizontal lines on the background of each panel that correspond to the interquartile range of each cell - short lines reflect consistent environment and long lines heterogeneous environment within the 1ha cell.

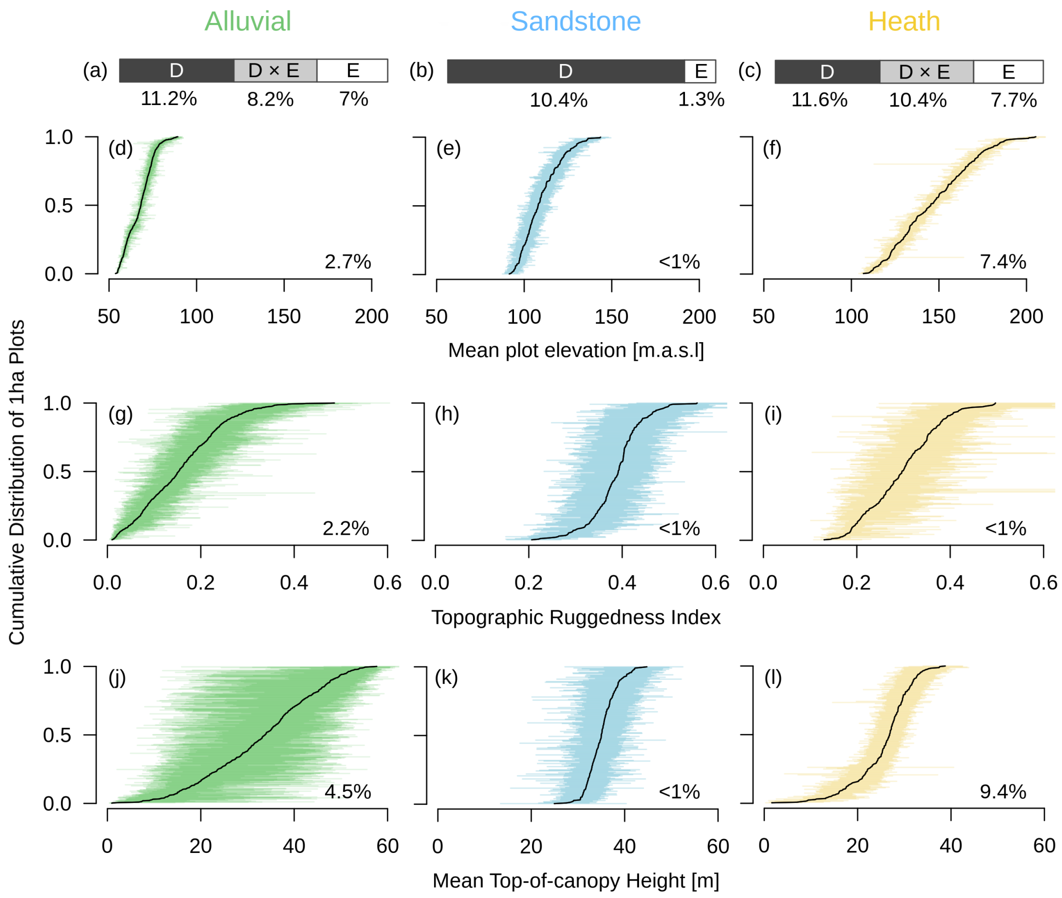

Supplemental Material, Figure S4: Environmental turnover (E) relates to compositional turnover as described in our GDM models (a-c). Cumulative distribution of mean 1ha cell environment is represented as a black line for the three ALS-derived environmental metrics that related to remotely-sensed β-diversity across the three forest types (d-l). Within-1ha cell environmental variability is represented by horizontal coloured lines, relating to the interquartile range of each cell.

Neutral modelling approach

Neutral simulations usually involve the following steps: (a) a random individual from a community is chosen to die and leave an empty space; (b) the replacement is chosen either to be a new species to the community (with some small probability ν, the per capita "speciation rate") or selected from propagules of existing individuals (with probability $1 - \nu$). Which propagule establishes in the space is determined using a dispersal kernel, representing the increased probability of nearby parents to provide offspring to the empty space. We used a two-parameter fat-tailed kernel (Clark *et al.*, 1999) for elevated probabilities of long-distance dispersal events occurring. Typical forward-time neutral models require many iterations to reach a dynamic equilibrium and quickly become computationally prohibitive at large spatial scales. We use a backward-time coalescence approach to avoid these issues (Rosindell *et al.*, 2008) producing equivalent results to forwards-time models with several orders of magnitude reduction in computation time. Coalescence allows for the full spatial structure of Borneo to be incorporated within the model - not just the Sepilok Forest Reserve - providing a more realistic, spatially accurate metacommunity. A single well-mixed habitat cell in our model was approximately 20m x 20m and contained a varying number of trees, depending on the density of the forest at that point. We used a combination of 50 dispersal and 7 speciation parameters for two sets of simulations, which resulted in 700 simulated virtual censuses over Sepilok. The range of parameters considered was beyond what can be expected biologically; the 7 speciation parameters were: 1^{-7}, 5^{-7}, 1^{-6}, 5^{-6}, 1^{-5}, 5^{-5}, 1^{-4}; We used latin-square sampling so that our 50 dispersal parameter combinations were evenly distributed across parameter space: measured in cell widths, σ ranged from 0.1 to 16 and τ ranged from 0.1 to 2. We used the laser scanning map of Sepilok to delineate the number of trees over the landscape using the approach of Coomes *et al.* (2017). The tree densities outside Sepilok were assumed to be the average of the tree density of each forest type.

We developed two types of simulations to compare with the observed landscape patterns in Sepilok. The interactions between trees were treated as entirely neutral in the first set of simulations with the whole of Borneo considered as a single arena where the offspring position is only limited by dispersal. We refer to those simulations as "forest-type-naive". In the second

set of simulations, we overlay forest type structure onto the arena and refer to them as "forest-type-aware". We used published soil maps of Sabah (from the Sabah Forestry Department) and Borneo (from FAO/UN) to define the extent of the forest types outside of our immediate study region (Fig. S5). The forest-type-aware simulations still treat interactions within forest type as neutral, but mixing between forest types is restricted. The ability of species to recruit outside their preferred forest type was calculated from 12-hectares of inventory data available for each forest type. A species with over 50% of its stems in a particular forest type was considered to have a preference for that forest type, and their mean abundance in each of the other forest types compared to the preferred site was used as recruitment coefficients. Parts of Borneo covered by environments not present in Sepilok were assumed to be a mix of the two non-preferred forest types when calculating recruitment. Overall, the forest-type-aware simulations incorporate forest type as an environmental filter in an otherwise neutral model. Allowing non zero density of all three niches in all environments permits dispersal between patches on a particular forest type that would otherwise be unreasonably restricted. In reality Sepilok is sufficiently isolated from primary forests that dispersal from outside its boundaries is no longer realistic, but its current assembly may well have been influenced by historic large forest extents that existed before the establishment of intensive tropical agriculture.

Note that we made no attempt to incorporate finer niche structure in our models. Dispersal and speciation parameters are key to the structure of diversity in neutral simulations. However, processes that operate below the 1-ha scale can manifest as apparently random fluctuations in β-diversity that are incorporated into this parameter, so we make no attempt to interpret it in a biological context, instead focusing on spatially autocorrelated diversity and the dispersal parameters that can reconstruct it. Patterns of spatial autocorrelation in biological diversity can be caused by limited seed dispersal (D), which results in close-together cells having more similar species composition than those further apart. It is also affected by environmental factors that may themselves be spatially autocorrelated (e.g. forest types occupy large patches with similar elevation or terrain ruggedness) so close-together cells are more likely to have the same forest type and environments (E$\times$D). In order to select the parameter sets that best fit the observed pattern, we compared GDM outputs between the modelled communities and the remotely-sensed β-diversity patterns. The neutral models reconstruct a spatially-explicit com-

munity that matches the boundaries of the Sepilok Forest Reserve. We can, therefore, analyse the virtual census using the same GDM approach as we did with the remote sensing data. GDMs output a set of splines that describe the contribution of the predictor variables to the measured dissimilarity; we compare those splines and select the models that most closely reconstruct the GDM models from the airborne survey. We compared the forest-type-aware models with the full extent of the spatially autocorrelated pattern (D + D$\times$E) of each forest type. We repeated the model selection, but this time requiring the models to only reconstruct the spatial structure that does not covary with the environmental component of the models (D component only).

Map of forest types across Borneo used for neutral simulations

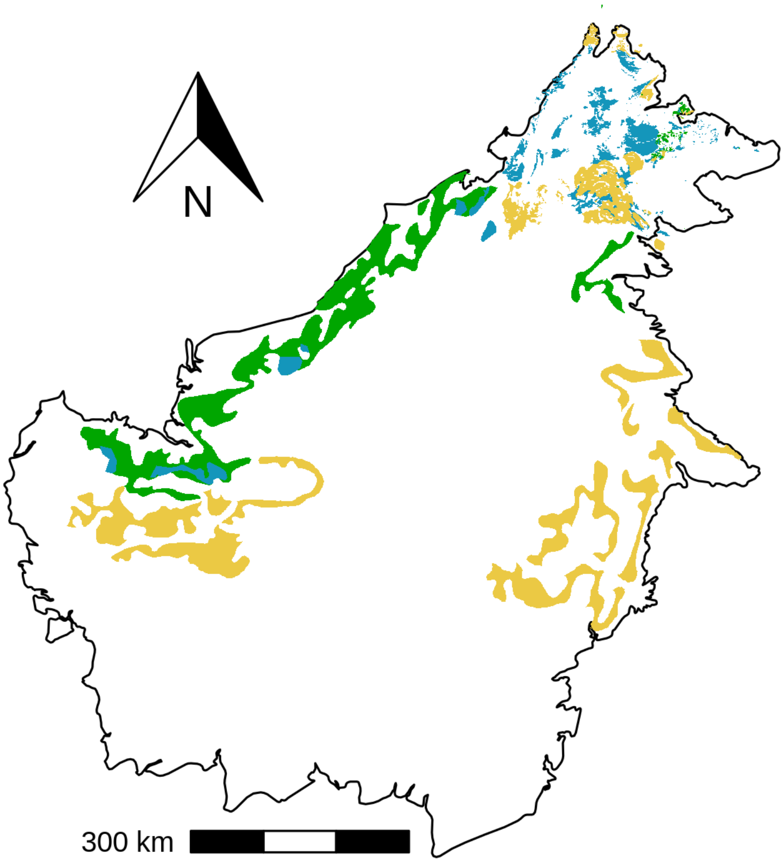

Supplemental Material, Figure S5: Distribution of the three floristic associations present in Sepilok (alluvial in green, sandstone in blue, heath in yellow) over the island of Borneo used in our neutral simulations. Distributions over the Malaysian state of Sabah are derived from a map courtesy of the Sabah Forestry Department based on a geological survey of the region. The forest type distributions outside of Sabah are based on a map provided by FAO/UN.

References

Berk, A., Anderson, G.P., Bernstein, L.S., Acharya, P.K., Dothe, H., Matthew, M.W., Adler-Golden, S.M., Chetwynd, J.H., Richtsmeier, S.C., Pukall, B. *et al.* (1999). *MODTRAN4 radiative transfer modeling for atmospheric correction*. vol. 3756.

Clark, J.S., Silman, M., Kern, R., Macklin, E. & HilleRisLambers, J. (1999). Seed dispersal near and far: patterns across temperate and tropical forests. *Ecology*, 80, 1475–1494.

Coomes, D.A., Dalponte, M., Jucker, T., Asner, G.P., Banin, L.F., Burslem, D.F., Lewis, S.L., Nilus, R., Phillips, O.L., Phua, M.H. *et al.* (2017). Area-based vs tree-centric approaches to mapping forest carbon in Southeast Asian forests from airborne laser scanning data. *Remote sensing of environment*, 194, 77–88.

Feilhauer, H., Asner, G.P., Martin, R.E. & Schmidtlein, S. (2010). Brightness-normalized partial least squares regression for hyperspectral data. *Journal of Quantitative Spectroscopy and Radiative Transfer*, 111, 1947–1957.

Jucker, T., Bongalov, B., Burslem, D.F., Nilus, R., Dalponte, M., Lewis, S.L., Phillips, O.L., Qie, L. & Coomes, D.A. (2018). Topography shapes the structure, composition and function of tropical forest landscapes. *Ecology Letters*.

Mantel, N. (1967). The detection of disease clustering and a generalized regression approach. *Cancer research*, 27, 209–220.

Rosindell, J., Wong, Y. & Etienne, R.S. (2008). A coalescence approach to spatial neutral ecology. *Ecological Informatics*, 3, 259–271.

Sculley, D. (2010). Web-scale k-means clustering. In: *Proceedings of the 19th international conference on World wide web*. ACM, pp. 1177–1178.
